# When Parathyroidectomy Should Be Indicated or Postponed in Adolescents With MEN1-Related Primary Hyperparathyroidism

**DOI:** 10.3389/fendo.2018.00597

**Published:** 2018-10-05

**Authors:** Francesca Marini, Francesca Giusti, Francesco Tonelli, Maria Luisa Brandi

**Affiliations:** Department of Surgery and Translational Medicine, University of Florence, Florence, Italy

**Keywords:** Multiple Endocrine Neoplasia Type 1, primary hyperparathyroidism, parathyroid adenomas, parathyroidectomy, MEN1 adolescent patients

## Abstract

Multiple Endocrine Neoplasia Type 1 (MEN1) is a rare inherited endocrine tumor syndrome principally affecting parathyroid glands, neuroendocrine tissues of the gastro-entero-pancreatic and thoracic tracts, and anterior pituitary, caused by germline inactivating mutations of the *MEN1* tumor suppressor gene. Primary hyperparathyroidism (PHPT) is usually the first clinical manifestation of the syndrome, normally manifesting during the third decade of life. Cases of affected children and adolescents have been described by the age of 5. Clinical characteristics and therapeutic management of MEN1 in adolescents have been described mainly by case reports. Only two studies on MEN1 patient series under the age of 22 years have recently been published. Given the scarcity of data and the lack of a consistent number of targeted studies, there are currently no specific guidelines available for children and adolescents with MEN1; diagnostic and therapeutic management is, thus, usually the same as for adult patients. Here, we report our experience with 19 adolescent MEN1 patients, developing MEN1-associated PHPT before the age of 20. Fourteen of them, manifesting hypercalcemic PHPT before the age of 20 underwent parathyroidectomy before the age of 25 to control calcemia. Parathyroid surgery restored normal calcemia in all the operated patients. No post-surgical nephrolithiasis has been reported after a mean of 12.0 ± 5.8 years of follow-up. Comparison between pre-surgical and post-surgical values of bone mineral density (BMD) in 2 patients evidenced an improvement of bone mass after parathyroid adenoma ablation. Two patients (14.28%) developed permanent post-surgical hypoparathyroidism.

## Introduction

Primary hyperparathyroidism (PHPT), caused by hyperplasia and/or adenomas of the parathyroids, is the most common (up to 100% after the age of 55 years) and, in the great majority of cases, the first clinical manifestation in Multiple Endocrine Neoplasia Type 1 (MEN1), a rare endocrine inherited multiple tumor syndrome caused by germline heterozygote inactivating mutations of the *MEN1* tumor suppressor gene.

PHPT in MEN1 manifests principally between 20 and 30 years of age, often remaining asymptomatic for years before being casually discovered during serum biochemical screening in index cases or being recognized by yearly biochemical dosage of parathyroid hormone (PTH) and serum calcium in *MEN1* mutation carriers. Cases of PHPT developed during childhood and adolescence have also been reported (1).

Since 1997, the application of the *MEN1* genetic test in members of mutated pedigrees has allowed the identification of asymptomatic carriers and a constant screening program for the early recognition of MEN1-associated biochemical signs and tumors (2). This has permitted increasingly frequent identification of PHPT cases during childhood and adolescence and treatment at a younger age.

Parathyroid surgery is the treatment of choice for MEN1-PHPT; surgical removal of abnormal gland/glands is the most effective and rapid cure for restoring normal parathyroid activity and quickly normalizing calcemia (3).

Available data about clinical characteristics and therapeutic management of MEN1-PHPT during childhood and adolescence are principally derived from very few single case reports. Only two studies (4, 5) on series of children and adolescents with MEN1 have recently been published. Both these studies confirmed PHPT as the most common and first clinical manifestation, also in young patients (58.3 and 73.8%, respectively), being asymptomatic in the great majority of cases (90.9 and 86%, respectively) and clinically diagnosed during yearly PHPT surveillance for MEN1 patients and/or for *MEN1* gene mutation carriers.

To date, no specific guidelines are available about the treatment of MEN1 and related PHPT in young individuals, and both medical and surgical therapies are usually the same as those applied to adults, according to the last international MEN1 guidelines (1). Currently, the right timing for surgery and the best surgical approach to treat MEN1-PHPT during the first two decades of life are still controversial.

In this paper, we describe our experience with MEN1 adolescent patients with PHPT, collected and followed-up through the Regional Referral Center for Inherited Endocrine Tumors of the Tuscany Region, at the “Azienda Ospedaliera-Universitaria Careggi,” Florence, Italy.

## Methods

### Patients

This study was approved by the Review Board of the “*Area Vasta Centro, Regione Toscana*” at the “*Azienda Ospedaliera-Universitaria Careggi*” (*Rif. CEAVC OSS 16.234*). All patients, or legal tutors for individuals under the age of 18, gave informed consent for data collection and analysis. All collected data were made appropriately anonymous and each patient was identified by a specific identification number; data were also analyzed as aggregates. Patients were collected and clinically followed up at the Ambulatory of the Regional Referral Center for Inherited Endocrine Tumors of the Tuscany Region from 1991 to 2017.

A total of 30 patients (17 females and 13 males), diagnosed with MEN1 before the age of 20, were collected in the database.

Genetic MEN1 diagnosis was made by Sanger's sequencing analysis of the *MEN1* gene. The entire coding region (exons 2-10) and splicing sites were screened in index cases. Target searching for the pedigree-specific mutation was performed in relatives of mutated patients.

Clinical diagnosis of MEN1 was made in the presence of at least one sign and/or symptom of one MEN1-associated tumor in familial cases, and of at least two signs and/or symptoms of one of the three principal MEN1-associated tumors in single cases (i.e., patient no. 20 presenting PHPT and insulinoma and patient no. 36 presenting PHPT and prolactinoma).

After MEN1 diagnosis (clinical or genetic), all patients were directed into the specific routine screening program for MEN1 tumors (1), including annual dosage of PTH (elevated PTH is considered over 7.6 pmol/l), total serum calcium (hypercalcemia is defined with value over 10.1 mg/dl), and calcium ion for the diagnosis of PHPT. Annual serum dosage of phosphorus was also performed (value <2.5 mg/dl, associated with elevated PTH, strengthens the diagnosis of PHPT).

Computed axial tomography (CAT) of kidneys were performed approximately every 2 years for monitoring the presence of renal calculi. Data about pre-operative dual X-ray absorptiometry (DEXA) evaluations of lumbar spine, femoral neck and total femur bone mineral density (BMD) were available only for two patients.

Pre-operatory imaging screening, by echo-color Doppler ultrasonography of the neck and cervical and mediastinal scintigraphy by Tc-99m sestamibi, were performed in all operated patients for the localization of parathyroids and the identification of possible super-numerary and/or ectopic glands, respectively.

All operated patients were followed-up by a yearly surveillance program for serum PTH and calcium to monitor post-surgical persistence and recurrences, as well as clinical and biochemical surveillance for MEN1 tumors (1). Post-surgical renal CAT was performed, approximately every 2 years, to monitor nephrolithiasis in all patients, with a mean of follow-up of 12.0 ± 5.8 years (range 3–23 years).

Three different surgical approaches were applied to our operated patients: (1) the resection of only the affected parathyroids, up to three glands (partial parathyroidectomy; PPTX), (2) the removal of three parathyroids and part of the fourth gland, the one appearing non-enlarged or hyperplastic (subtotal parathyroidectomy; STPTX), and (3) the complete ablation of all parathyroids (total parathyroidectomy, TPTX) with auto-transplantation of normal parathyroid tissue in the non-dominant forearm.

Intra-operative dosage of PHT was performed during all interventions to monitor PTH levels, to confirm the ablation of all affected glands.

## Results

Our database included a total of 30 patients diagnosed with MEN1 before the age of 20 (mean age at first MEN1 diagnosis 11.87 ± 4.92 years; range 0–20 years), all, but one, with a positive *MEN1* genetic test. Nineteen were first diagnosed by the genetic test, while 11 had a MEN1 clinical diagnosis before the performance of the genetic test (individuals collected before the development of *MEN1* testing).

Annual biochemical evaluation of PTH, total serum calcium and calcium ion, starting by the time of MEN1 diagnosis, allowed the recognition of PHPT at its onset.

Ten of 30 subjects (33.33%), aged <20 years at the time of this study, have not shown any sign or symptom of MEN1; they were diagnosed with MEN1 only by the genetic test. One female patient developed a prolactinoma at the age of 13, but she had not manifested any sign of PHPT at the time of this study (age of 24 years). They are all still under clinical surveillance for MEN1 tumors (1).

Nineteen individuals (13 females and 6 males; 19/30; 63.33%) showed elevated serum level (over 7.6 pmol/L; normal range 1.3–7.6 pmol/L) of PTH before the age of 20 (mean age of first recognition of elevated PTH serum level 16.32 ± 2.15 years; range 12–19 years). They included 2 MEN1 single cases (1 with an identified *MEN1* gene mutation, and 1 negative to *MEN1* sequencing analysis but clinically diagnosed with MEN1), and 17 MEN1 familial cases from 13 different pedigrees (16 with an identified *MEN1* gene mutation and 1 with two different *MEN1* mutations, both located on the same gene allele). Three of 19 patients were referred to our Endocrine Referral Center as affected index cases [2 MEN1 single cases (1 with a negative *MEN1* genetic test but clinically diagnosed as MEN1) and 1 MEN1 familial case (with both genetic and clinical MEN1 diagnosis)]. The other 16 individuals were relatives of MEN1 index cases, and they were diagnosed by the clinical recognition of MEN1 signs and symptoms as members of affected MEN1 families and/or by the genetic test (in families with a previously identified *MEN1* gene mutation).

Five of these 19 patients (26.32%) (mean age at first recognition of elevated PTH serum level 16.20 ± 2.63 years; range 12–19 years), were still normocalcemic and asymptomatic at the time of this study and did not undergo parathyroid surgery. Main individual clinical characteristics of non-operated patients are reported in Table 1.

**Table 1:** Main individual clinical characteristics of MEN1 individual with normocalcemic elevated PTH level, who did not undergo PTX.

**Patient's numeric code**	**Gender**	**MEN1 single case (S) or familial form (F)**	**Age at MEN1 genetic diagnosis**	**Age at MEN1 clinical diagnosis**	**First MEN1 clinical manifestation**	**Age at PHPT clinical diagnosis**	**Symptomatic (S) or asymptomatic (A) PHPT**	**Normocalcemia (N) or hypercalcemia (H)**	**Osteopenia or osteoporosis**	**Nephrolithiasis**	**Other MEN1 neuroendocrine tumors at the time of PHPT diagnosis**	**Notes**
42	M	F	16	15	PHPT/PRLoma	15	A	N	n.a.	NO	PRLoma	
43	F	F	10	16	PHPT	16	A	N	n.a.	NO	NO	Patient refused to undergo PTX and she has been treated with cinacalcet since 2007
44	M	F	7	19	PHPT/PRLoma	19	A	N	n.a.	NO	PRLoma	
45	F	F	9	19	PHPT	19	A	N	n.a.	NO	NO	
46	F	F	12	12	PHPT	12	A	N	n.a.	NO	NO	

*PHPT, primary hyperparathyroidism; PTH, parathyroid hormone; PTX, parathyroidectomy; PRLoma, prolactinoma; n.a., non-available. Patients 43 and 44 belong to one family (brother and sister) and they are also brothers of patient 9 (Table 2)*.

The other fourteen PHPT patients (14/19; 73.68%) were diagnosed with PHPT (mean age at PHPT clinical diagnosis 16.36 ± 1.95 years; range 13–19 years), manifested hypercalcemia (all of them >11 mg/dL of total serum calcium) and underwent parathyroid surgery. Twelve (63.16%) presented hypercalcemia before the age of 20 and were operated under the same age (mean age at surgical intervention 17.08 ± 2.18 years; range 13–20 years). The other two patients were operated, respectively, at the ages of 22 and 25 years. For the first one (case 3, Table 2), the decision for surgery was delayed up to the manifestation of hypercalcemia at the age of 22. The second one (case 9, Table 2) was treated (after developing the first sign of hypercalcemia at the age of 21) for about 3 years with cinacalcet, before the decision for surgery because of a severe osteoporosis at the age of 25.

**Table 2:** Main individual clinical characteristics of PHPT-affected MEN1 patients at the time of parathyroid surgery.

**Patient's numeric code**	**Gender**	**MEN1 single case (S) or familial form (F)**	**Age at MEN1 genetic diagnosis**	**Age at MEN1 clinical diagnosis**	**First MEN1 clinical manifestation**	**Age at PHPT clinical diagnosis**	**Symptomatic (S) or asymptomatic (A) PHPT**	**Normocalcemia (N) or hypercalcemia (H)**	**Osteopenia or osteoporosis**	**Nephrolithiasis**	**Other MEN1 neuroendocrine tumors at the time of PTX**	**Notes**
1	F	F	20	15	PRLoma	15	S	H	Osteopenia (lumbar spine and femur)	NO	PRLoma	
3	M	F	13	15	PHPT	15	A	H	n.a.	NO	NO	
9	M	F	13	12	PRLoma	16	S	H	Severe osteoporosis (lumbar spine and femur)	NO	PRLoma	Fragility fracture of the distal epiphysis of radial bone Vitamin D deficiency Unresectable macroPRLoma poorly responsive to pharmacological therapy Severe deficiency of testosterone (pharmacological substitutive therapy)
10^*^	F	F	19	17	Insulinoma	18	A	H	n.a.	NO	Insulinoma	Surgical resection of insulinoma was performed 1 year before PTX
12	F	F	19	17	PHPT (kidney colic and stones)	18	S	H	n.a.	YES	NO	
16	F	F	29	19	PRLoma	19	A	H	n.a.	NO	PRLoma	
18	F	F	13	14	PHPT (kidney colic)	14	S	H	n.a.	YES	NO	
20^*^	F	S	28	20	PHPT	15	S	H	n.a.	YES	Insulinoma	Insulinoma was detected and surgically resected between the first and the second PTX
22	F	F	19	18	PHPT (kidney stones)	18	S	H	n.a.	YES	NO	
26	M	F	25	18	PHPT	18	A	H	n.a.	NO	PRLoma, insulinoma	Insulinoma was surgically resected the same year of PTX
27	F	F	8	14	PHPT	14	A	H	n.a.	NO	NO	
29	M	F	11	14	PRLoma	17	A	H	n.a.	NO	PRLoma	
36^*^	F	S	Negative *MEN1* sequencing test	13	PRLoma	13	A	H	n.a.	NO	PRLoma	
38	F	F	34	19	PHPT	19	A	H	n.a.	NO	NO	Total thyroidectomy for Hurtle cell carcinoma at the time of first PTX

PHPT, primary hyperparathyroidism; PTH, parathyroid hormone; PTX, parathyroidectomy; PRLoma, prolactinoma; n.a., non-available. Patients indicated with the ^*^ were referred to our Center as MEN1 affected index cases. Patients 10 and 12 belong to one family (two sisters); patients 26 and 27 to one family (26 is uncle of 27). Patient 9 is brother of patients 43 and 44 (Table 1).

The main individual clinical characteristics at the time of parathyroid surgery of our operated MEN1 patients are summarized in Table 2.

Pre-operatory imaging screenings allowed us to recognize ectopic parathyroids in two patients (2/14; 14.29%) (cases 29 and 36 in Tables 2, 3). In particular, scintigraphy permitted us to identify an ectopic adenomatous gland in the mediastinum aorto-pulmonary window of a patient 29 (Tables 2, 3).

**Table 3:** Main characteristics of parathyroid surgery in our MEN1 adolescent patients (up to 20 years of age at the time of PHPT clinical diagnosis).

**Patient's numeric code**	***MEN1* gene mutation**	**Age at PHPT clinical diagnosis**	**Age at first PTX**	**Surgical intervention type**	**Normal parathyroid tissue auto-re-implantation during first PTX**	**PHPT persistence after the first PTX**	**PHPT recurrence after the first PTX**	**Second PTX (re-intervention)**	**Age at second PTX**	**Normal parathyroid tissue auto-re-implantation during second PTX**	**Post-surgical hypoparathyroidism**
1	Missense Gly163Arg, exon 3	15	20	TPTX	Yes	No	No	No	n.a.	n.a.	Yes
3	Nonsense Gln508Stop, exon 10	15	22	TPTX	Yes	No	No	No	n.a.	n.a.	Yes
9	Splicing g.893+1(G>A), intron 4	16	25	STPTX (PHPT was previously treated with cinacalcet for 3 years before surgery)	No	No	No	No	n.a.	n.a.	No
10*	Nonsense Arg460Stop, exon 10	18	18	TPTX	Yes	No	No	No	n.a.	n.a.	No
12	Nonsense Arg460Stop, exon 10	18	18	TPTX	Yes	No	No	No	n.a.	n.a.	No
16	Frameshift g.1364delC, exon 9	19	19	TPTX	Yes	No	Yes	TPTX (forearm reimplant)	30	no	No
18	Missense Val196Gly, exon 3	14	15	PPTX (1 gland)	No	No	Yes	PPTX (1 gland)	19	n.a.	No
20*	Missense His181Asp, exon 3	15	15	PPTX (1 gland)	No	No	Yes	TPTX	29	yes	No
22	Missense Trp220Arg, exon 4	18	18	PPTX (2 glands)	No	Yes	No	PPTX	21	no	No
26	Frameshift g.359del(GTCT), exon 2	18	19	TPTX	Yes	No	No	No	n.a.	n.a.	No
27	Frameshift g.359del(GTCT), exon 2	14	14	TPTX	Yes	No	No	No	n.a.	n.a.	No
29	Double mutation on the same MEN1 allele: Missense Leu249Pro, exon 4; Frameshift g.1181delC, exon 8	17	17	PPTX (1 ectopic gland)	No	No	No	No	n.a.	n.a.	No
36*	Negative *MEN1* sequencing test	13	13	TPTX (included 2 additional ectopic glands)	Yes	No	No	No	n.a.	n.a.	No
38	Frameshift g.1656insC, exon 10	19	19	STPTX	No	No	Yes	TPTX	24.	no	No

PHPT, primary hyperparathyroidism; PTX, parathyroidectomy; PPTX, partial parathyroidectomy; STPTX, subtotal parathyroidectomy; TPTX, total parathyroidectomy; n.a., non-applicable. Patients indicated with the * were referred to our Center as MEN1 affected index cases. Patients 10 and 12 belong to one family (two sisters); patients 26 and 27 to one family (26 is uncle of 27).

The 14 operated patients presented a mean gap between PTHP clinical diagnosis and surgery of 1.57 ± 2.95 years (range 0–9 years). Ten/fourteen (71.43%) underwent PTX the same year as PHPT clinical diagnosis (0-year gap). At the time of parathyroid surgery, eight hypercalcemic patients (8/14; 57.14%) were still asymptomatic for PHPT, while six (6/14; 42.86%) also manifested PHPT-related symptoms (four with kidney stones, one osteopenia, and one severe osteoporosis with fragility fracture of the distal epiphysis of radial bone). No correlation was reported between the presence of hypercalcemia and/or value of elevated calcium level and the manifestation of a symptomatic PHPT, both in terms of nephrolithiasis and renal colic and bone mass reduction.

Interestingly, six patients presented an active prolactinoma at the time of PTX. One patient affected by insulinoma underwent pancreatic surgery a year before PTX (case 10); another patient was detected with an insulinoma and underwent pancreatic resection between the first PTX and the re-intervention (case 20) (Table 2).

TPTX was the most commonly performed intervention (8 cases; 57.14%) in all cases in association with normal parathyroid tissue autograft (8–10 pieces of about 1 mm^3^ in volume of the parathyroid gland appearing as non-adenomatous) in the brachio-radial muscle of the non-dominant forearm, followed by PPTX (4 cases; 28.57%) and STPTX (2 cases; 14.29%).

Five patients (35.71%) underwent a second PTX (mean age of 7.40 ± 4.32 years after the first surgical intervention; range 3–14 years), one for PHPT persistence (3 years after the first PPTX) and four for recurrences (mean age of 8.50 ± 4.15 years after the first surgical intervention; range 4–14 years). One re-intervention was TPTX secondary to a precedent PPTX (20%), one was a TPTX secondary to a precedent STPTX (20%), two were PPTX secondary to a precedent PPTX (40%), and one was a TPTX to totally remove adenoma recurrence at the forearm re-implant, subsequent to a precedent TPTX (20%).

Two patients (14.29%) developed post-surgical permanent hypoparathyroidism, both after TPTX (25% of performed TPTX).

Main characteristics of parathyroid surgery in our 14 MEN1 operated adolescents are summarized in Table 3.

No patients developed nephrolithiasis during post-surgical follow-up; the four patients with pre-operatory renal calculi manifest no post-operatory variation in calculi number and/or size.

Available data regarding pre- and post-operatory DEXA measurement of BMD values at lumbar spine, femoral neck and total femur are resumed in Table 4.

**Table 4:** Pre-operatory and post-operatory DEXA measurement of BMD in operated patients.

**Patient**	**PHPT surgery (date)**	**Pre-operatory DEXA (date)**	**Lumbar spine (L1–L4) BMD**	**Lumbar spine *T-Score***	**Lumbar spine *Z-Score***	**Femoral neck BMD**	**Femoral neck *T*-score**	**Femoral neck *Z*-score**	**Total femur BMD**	**Total femur *T*-score**	**Total femur *Z*-score**	**First post-operatory DEXA (date)**	**Lumbar spine (L1–L4) BMD**	**Lumbar spine *T*-score**	**Lumbar spine *Z*-score**	**Femoral neck BMD**	**Femoral neck *T*-score**	**Femoral neck *Z*-score**	**Total Ffmur BMD**	**Total femur *T*-score**	**Total femur *Z*-score**
1	2013	2013	**0.923**	−**1.3**	−**1**	**0.714**	−**1.8**	−**1.8**	**0.802**	−**1.4**	−**1.4**	2014	**0.998**	−**0.4**	−**0.3**	**0.785**	−**0.6**	−**0.6**	**0.876**	−**0.5**	−**0.5**
3	2007	n.a.	n.a.	n.a.	n.a.	n.a.	n.a.	n.a.	n.a.	n.a.	n.a.	2016	1.145	0.5	0.5	0.898	–0.2	0	1.154	0.8	0.9
9	2013	2013	**0.61**	−**4.4**	−**4.4**	**0.563**	−**2.7**	−**2.7**	**0.659**	−**2.5**	−**2.5**	2015	**0.658**	−**3.9**	−**3.9**	**0.608**	−**2.4**	−**2.3**	**0.713**	−**2.1**	−**2.1**
10	2001	n.a.	n.a.	n.a.	n.a.	n.a.	n.a.	n.a.	n.a.	n.a.	n.a.	2016	0.971	–0.7	–0.7	0.675	–1.6	–1.4	0.807	–1.4	–1.1
12	2001	n.a.	n.a.	n.a.	n.a.	n.a.	n.a.	n.a.	n.a.	n.a.	n.a.	2016	0.89	–1.4	–1.4	0.651	–1.8	–1.7	0.736	–1.7	–1.6
16	2001	n.a.	n.a.	n.a.	n.a.	n.a.	n.a.	n.a.	n.a.	n.a.	n.a.	2013	0.979	–1.7	–2	0.881	–0.8	–1	1.001	0	–0.2
18	2010	n.a.	n.a.	n.a.	n.a.	n.a.	n.a.	n.a.	n.a.	n.a.	n.a.	2016	1.063	0.1	0.4	0.873	0.2	0.2	0.975	0.1	0.1
20	2006	n.a.	n.a.	n.a.	n.a.	n.a.	n.a.	n.a.	n.a.	n.a.	n.a.	2017	0.922	–1.1	–0.9	0.767	–0.7	–0.5	0.851	–0.7	–0.6
22	2005	n.a.	n.a.	n.a.	n.a.	n.a.	n.a.	n.a.	n.a.	n.a.	n.a.	2013	0.926	–1.1	–1	0.77	–0.7	–0.7	0.872	–0.6	–0.6
26	1994	n.a.	n.a.	n.a.	n.a.	n.a.	n.a.	n.a.	n.a.	n.a.	n.a.	2013	1.048	–0.4	–0.3	0.713	–1.6	–1.2	0.888	–1	–0.8
27	2006	n.a.	n.a.	n.a.	n.a.	n.a.	n.a.	n.a.	n.a.	n.a.	n.a.	2016	1.272	2.3	2.4	0.953	1	1	1.138	1.6	1.6
29	2014	n.a.	n.a.	n.a.	n.a.	n.a.	n.a.	n.a.	n.a.	n.a.	n.a.	n.a.	n.a.	n.a.	n.a.	n.a.	n.a.	n.a.	n.a.	n.a.	n.a.
36	1999	n.a.	n.a.	n.a.	n.a.	n.a.	n.a.	n.a.	n.a.	n.a.	n.a.	n.a.	n.a.	n.a.	n.a.	n.a.	n.a.	n.a.	n.a.	n.a.	n.a.
38	2000	n.a.	n.a.	n.a.	n.a.	n.a.	n.a.	n.a.	n.a.	n.a.	n.a.	2016	0.969	–0.7	–0.6	0.734	–1	–0.9	0.846	–0.8	–0.7
**Patient**	**PHPT surgery (date)**	**Second post-operatory DEXA (date)**	**Lumbar spine (L1–L4) BMD**	**Lumbar spine** ***T*****-score**	**Lumbar spine** ***Z*****-score**	**Femoral neck BMD**	**Femoral neck** ***T*****-score**	**Femoral neck** ***Z*****-score**	**Total femur BMD**	**Total femur** ***T*****-score**	**Total femur** ***Z*****-score**	**Third post-operatory DEXA (date)**	**Lumbar spine (L1–L4) BMD**	**Lumbar spine** ***T*****-score**	**Lumbar spine** ***Z*****-score**	**Femoral neck BMD**	**Femoral neck** ***T*****-score**	**Femoral neck** ***Z*****-score**	**Total femur BMD**	**Total femur** ***T*****-score**	**Total femur** ***Z*****-score**
1	2013	n.a.	n.a.	n.a.	n.a.	n.a.	n.a.	n.a.	n.a.	n.a.	n.a.	n.a.	n.a.	n.a.	n.a.	n.a.	n.a.	n.a.	n.a.	n.a.	n.a.
3	2007	n.a.	n.a.	n.a.	n.a.	n.a.	n.a.	n.a.	n.a.	n.a.	n.a.	n.a.	n.a.	n.a.	n.a.	n.a.	n.a.	n.a.	n.a.	n.a.	n.a.
9	2013	2016	**0.706**	−**3.5**	−**3.5**	**0.6**	−**2.4**	−**2.3**	**0.715**	−**2.1**	−**2.1**	n.a.	n.a.	n.a.	n.a.	n.a.	n.a.	n.a.	n.a.	n.a.	n.a.
10	2001	n.a.	n.a.	n.a.	n.a.	n.a.	n.a.	n.a.	n.a.	n.a.	n.a.	n.a.	n.a.	n.a.	n.a.	n.a.	n.a.	n.a.	n.a.	n.a.	n.a.
12	2001	n.a.	n.a.	n.a.	n.a.	n.a.	n.a.	n.a.	n.a.	n.a.	n.a.	n.a.	n.a.	n.a.	n.a.	n.a.	n.a.	n.a.	n.a.	n.a.	n.a.
16	2001	2014	0.94	–2	–2.1	0.877	–0.9	–1	1.009	0.1	–0.1	2016	0.859	–1.7	–1.3	0.742	–1	–0.6	0.887	–0.5	–0.2
18	2010	n.a.	n.a.	n.a.	n.a.	n.a.	n.a.	n.a.	n.a.	n.a.	n.a.	n.a.	n.a.	n.a.	n.a.	n.a.	n.a.	n.a.	n.a.	n.a.	n.a.
20	2006	n.a.	n.a.	n.a.	n.a.	n.a.	n.a.	n.a.	n.a.	n.a.	n.a.	n.a.	n.a.	n.a.	n.a.	n.a.	n.a.	n.a.	n.a.	n.a.	n.a.
22	2005	2014	0.926	–1.1	–1.1	0.81	–0.4	–0.3	0.897	–0.4	–0.4	2015	0.946	–0.9	–0.9	0.824	–0.2	–0.2	0.883	–0.5	–0.5
26	1994	2015	1.04	–0.5	–0.4	0.716	–1.6	–1.1	0.982	–0.3	–0.2	n.a.	n.a.	n.a.	n.a.	n.a.	n.a.	n.a.	n.a.	n.a.	n.a.
27	2006	n.a.	n.a.	n.a.	n.a.	n.a.	n.a.	n.a.	n.a.	n.a.	n.a.	n.a.	n.a.	n.a.	n.a.	n.a.	n.a.	n.a.	n.a.	n.a.	n.a.
29	2014	n.a.	n.a.	n.a.	n.a.	n.a.	n.a.	n.a.	n.a.	n.a.	n.a.	n.a.	n.a.	n.a.	n.a.	n.a.	n.a.	n.a.	n.a.	n.a.	n.a.
36	1999	n.a.	n.a.	n.a.	n.a.	n.a.	n.a.	n.a.	n.a.	n.a.	n.a.	n.a.	n.a.	n.a.	n.a.	n.a.	n.a.	n.a.	n.a.	n.a.	n.a.
38	2000	n.a.	n.a.	n.a.	n.a.	n.a.	n.a.	n.a.	n.a.	n.a.	n.a.	n.a.	n.a.	n.a.	n.a.	n.a.	n.a.	n.a.	n.a.	n.a.	n.a.

*DEXA, dual X-ray absorptiometry; n.a., non-available. Data in bold are referred to patients for whom both a pre-operatory and a post-operatory DEXA analyses are available*.

No association has been found between type and localization of *MEN1* mutations and PHPT clinical presentation and/or response to surgery.

## Discussion

Parathyroid surgery represents the most effective therapy for PHPT in MEN1 patients, granting the rapid restoration of both normal PTH secretion and serum calcium level. A recent study by Goswami et al. (6) evidenced that the cure of PHPT, in adults with MEN1, significantly improved the health-related quality of life regarding fatigue, depression, anxiety, and social life, suggesting that parathyroid surgery should be prioritized in these patients. Currently, the choice of the correct timing and the most decisive type of PTX in MEN1 is still debated, both for adults and, even more, for children and adolescents.

In general, studies on the clinical features of pediatric and adolescent PHPT, both sporadic and inherited non-syndromic or syndromic forms, are very limited, and comparisons between series of Caucasian young and adult patients are still lacking. A very recent study has compared 59 pediatric Chinese PHPT patients (only 2 of them bearing a germline *MEN1* mutation) with 118 adult Chinese PHPT patients (7). The study has demonstrated that pediatric PHPT can be successfully treated by PTX like adults.

Two recent studies analyzed series of adolescent patients with MEN1 (4, 5). The study of Goudet (5) reported that 60.7% of their PHPT young patients (under the age of 21 years) underwent parathyroid surgery; 67.57% were operated by a PPTX/less than subtotal parathyroidectomy of only the affected glands and 32.43% by a STPTX; no TPTX was performed in their patients. Post-surgical results, in restoring normal calcemia, inducing transitory and/or permanent hypocalcemia, and rate of persistence and recurrences, were comparable with data published regarding MEN1 adult patients (8–10). Vannucci et al. (4) did not report any data about parathyroid surgery in their series.

Neither guidelines nor surgeon consensus statements are available at the moment regarding when and how PTX should be performed in children and adolescents with MEN1-related PHPT. Targeted studies to evaluate the effects of early PTX in adolescents with MEN1-PHPT, both as positive clinical outcomes and improvement of general quality of life, are surely required.

Persistent excess of PTH is responsible for increased bone resorption and reduction of calcium deposition on bone tissue, leading to a pathological loss of cortical and trabecular bone and early osteopenia/osteoporosis with risk of fragility fractures both in sporadic PHPT (11) and MEN1-related PHPT (12, 13). MEN1-associated PHPT, which develops in young adults and also in children and adolescents, can also severely alter the establishment of bone mass peak and be responsible for a higher risk of severe osteoporosis and fragility fractures (12, 13). Excessive PTH-dependent bone loss is reversible; restoration of normal PTH level is fundamental to block abnormal bone resorption and recover healthy BMD and bone mass. Early surgery is the most effective way to preserve bone mass in patients with MEN1-PHPT (12, 14).

Thus, the decision for PTX timing and surgical approach in MEN1 adolescents has to take into account the ratio between benefits (in terms of clinical outcomes and general health-related quality of life) and the risk of post-surgical complications (i.e., permanent hypoparathyroidism in extensive surgery such as STPTX and TPTX, or recurring laryngeal nerve damages subsequent to neck re-operation after PPTX), and should be driven on the basis of patient personal clinical characteristics (i.e., hypercalcemia, BMD value, kidney functionality and presence of recurrent renal calculi, presence of other neuroendocrine tumors and related hormone-syndrome). Some clinical characteristics may to lead a decision, both for adult and young patients, for surgical intervention: (1) hypercalcaemia [to treat symptoms (kidney stones, reduced bone calcium apposition, nausea, anxiety, fatigue, lethargy, depression, confusion, anorexia, vomiting, constipation, etc.) and to prevent consequences of a prolonged untreated hypercalcaemia, such as kidney insufficiency, osteoporosis, fragility fractures, neuromuscular affections and cardiovascular morbidity], (2) symptomatic PHPT (i.e., nephrolithiasis and/or osteopenia/osteoporosis; ablation of tumors demonstrated to prevent future incidence of renal calculi and to rapidly restore BMD value), (3) presence of an active gastrinoma [i.e., elevated serum calcium level increases gastrin secretion of gastrinoma, exacerbating Zollinger-Ellison syndrome (ZES) and increasing the risk of peptic ulcers] (15). The presence of an active insulinoma is an absolute indication to postpone PTX until after curing the hypoglycemia.

Interventions performed by experienced surgeons, within multidisciplinary referral centers for inherited endocrine tumors (in which a relatively high number of MEN1 cases are operated every year), surely grant better therapeutic results and positive post-surgical outcomes, both in terms of reduction of complications and decrease of rate of persistence/recurrences.

Indication for parathyroid surgery for all our operated patients was the presence of elevated PTH (over 7.6 pmol/L) associated with hypercalcemia over the value of 11 mg/dL of total serum calcium, both for symptomatic and asymptomatic PHPT.

**Figure 1:**
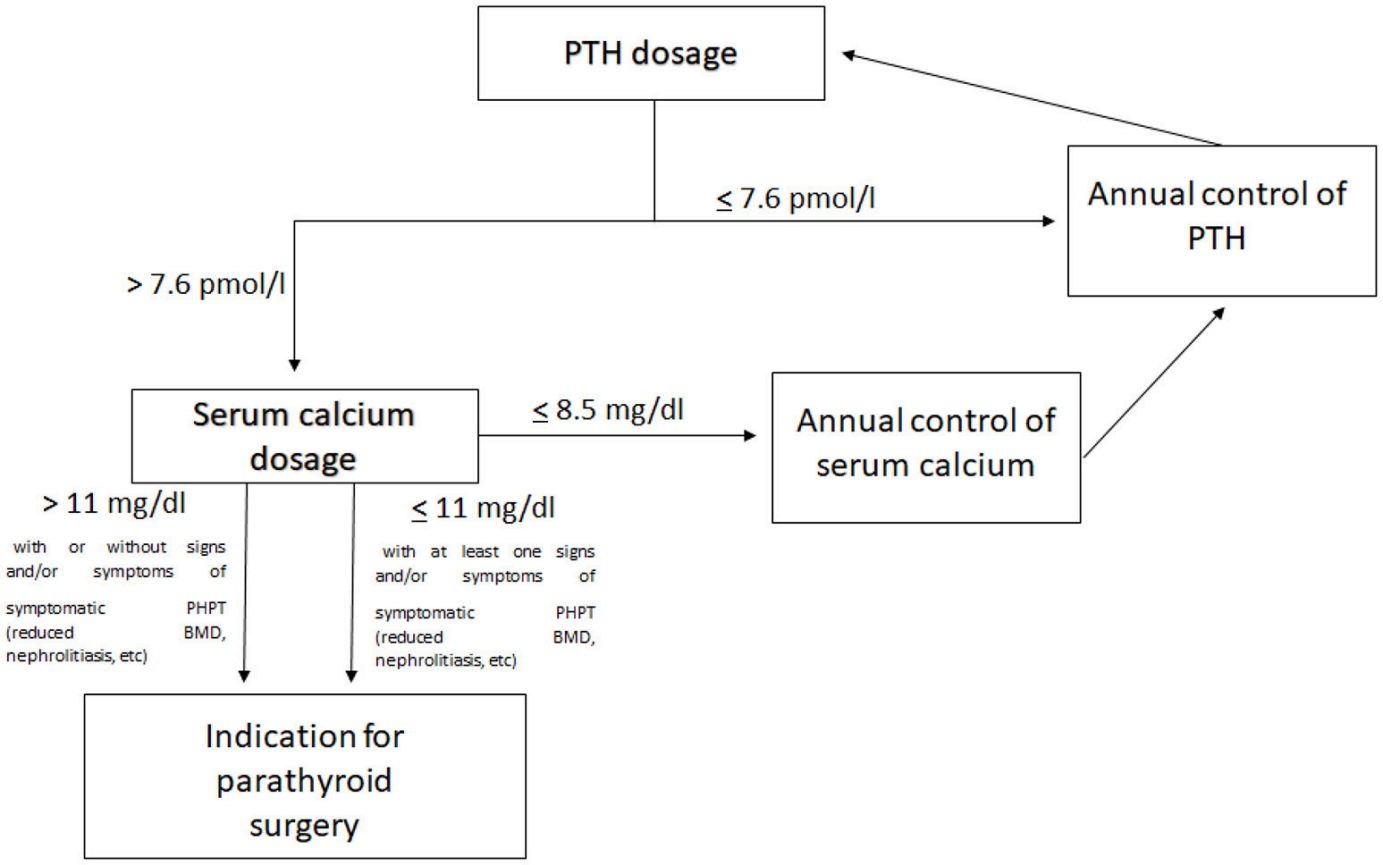
Proposed PHPT workup in MEN1 patients.

A suggested workup for the management of PHPT in MEN1 patients, including children and adolescents, is represented in Figure 1.

Given the genetic predisposition in *MEN1* mutated individuals of every parathyroid cell to lose the second wild type copy of the *MEN1* gene and to randomly develop multiple hyperplasia/adenoma (affecting, asynchronously, and asymmetrically, all four glands during the patient's lifetime), and because of the common (up to 30%) presence of supernumerary and/or ectopic parathyroids (16), all our patients were followed-up by a yearly surveillance program for serum PTH and calcium to monitor post-surgical persistence and recurrences. In our young patients, surgical intervention restored normal values of PTH and serum calcium (both as calcium ion and total serum calcium).

Only one case of PHPT persistence was reported (after a PPTX) to require a second PPTX 3 years after the first surgery. Total rate of PHPT persistence and recurrences (5/14; 35.71%) in our series is comparable to that reported in literature for MEN1 patients (17). In our series, PPTX presented a high rate of persistence (1/4; 25%) and recurrence (2/4; 50%), while TPTX showed recurrence in only one case (1/8; 12.5%). We reported two cases of permanent hypoparathyroidism, both after TPTX. Our data indicate that the TPTX approach grants a longer recurrent PHPT-free period, and a lower risk of re-intervention (which most of the time is performed at the transplantation site in the forearm and not at the neck). The risk of permanent hypoparathyroidism is higher with TPTX, but it could be mitigated over the years.

Pre-operatory and a post-operatory DEXA measurements of BMD were available only for two individuals. One of them, presenting pre-operatory hypercalcemia, lumbar spine *T*-score −1.3 and *Z*-score −1.0, femoral neck T and *Z*-scores −1.8, total femur T and *Z*-scores −1.4, showed to have recovered a normal bone mass, as assessed by a lumbar and femoral DEXA evaluation 4 years after surgery (lumbar spine *T*-score −0.4, *Z*-score −0.3; femoral neck T and *Z*-scores −0.6; total femur T and *Z*-scores −0.5). The other one, showing a pre-operative borderline normocalcemia (controlled by cinacalcet) but a severe osteoporosis (lumbar spine T and *Z*-scores −4.4; femoral neck T and *Z*-scores −2.7; total femoral T and *Z*-scores −2.5) associated with a fragility fracture, underwent a STPTX and did not manifest any PHPT persistence or recurrence up to 4 years after surgery. However, he showed the persistence of a severe osteoporosis (in presence of a post-operative normocalcemic status and a correct PTH level) at two (lumbar spine T and *Z*-scores −3.9; femoral neck *T*-score −2.4 and *Z*-score −2.3; total femoral T and *Z*-scores −2.1) and three (lumbar spine T and *Z*-scores −3.5; femoral neck *T*-score −2.4 and *Z*-score −2.3; total femoral T and *Z*-scores −2.1) years after parathyroid surgery. These data suggested that the severely reduced BMD in this very young MEN1 patient was, presumably, not due to PHPT only. Indeed, this young male presented, by the age of 12, an active non-resectable macroprolactinoma poorly responsive to treatment with high dosage of cabergoline, inducing severe deficiency of testosterone and being, presumably, a major cause of the highly reduced bone mass.

Several *MEN1* mutations in our patients neither influenced the clinical PHPT phenotype nor had any role in directing the choice of individual PTX, both in terms of timing and type of intervention. Rate of PHPT persistence and recurrences were demonstrated to be dependent only on the type of parathyroid surgery and not on the specific *MEN1* gene mutation.

Our results confirmed PTX as the optimal treatment of PHPT, presenting, in case of hypercalcemia > 11 mg/dl, a good benefit/risk balance, even in adolescents. Even if early PTX may predispose young patients to earlier persistence/recurrences of PHPT and the need of reoperation, a delay in surgical removal of parathyroid tumors may worsen clinical consequences of long-term hypercalcemia.

## Author contributions

FM analyzed patients' data, wrote the manuscript, and designed the tables. FG collected patients' data, created patients' database and helped in writing the manuscript and designed the tables. FT performed parathyroid surgery and helped in writing the manuscript. MB designed the study and revised the final version of the manuscript.

### Conflict of interest statement

The authors declare that the research was conducted in the absence of any commercial or financial relationships that could be construed as a potential conflict of interest.

## References

[1] Thakker RV Newey PJ Walls GV Bilezikian J Dralle H Ebeling PR. Clinical practice guidelines for multiple endocrine neoplasia type 1 (MEN1). J Clin Endocrinol Metab. (2012) 97:2990–3011. 10.1210/jc.2012-1230 22723327

[2] van Leeuwaarde RS van Nesselrooij BP Hermus AR Dekkers OM de Herder WW van der Horst-Schrivers AN Impact of delay in diagnosis in outcomes in MEN1: results from the Dutch MEN1 Study Group. J Clin Endocrinol Metab. (2016) 101:1159–65. 10.1210/jc.2015-3766 26751192

[3] Giusti F Tonelli F Brandi ML. Primary hyperparathyroidism in multiple endocrine neoplasia type 1: when to perform surgery? Clinics (2012) 67 (Suppl. 1):141–4. 10.6061/clinics/2012(Sup01)23 22584719 PMC3328829

[4] Vannucci L Marini F Giusti F Ciuffi S Tonelli F Brandi ML. MEN1 in children and adolescents: data from patients of a regional referral center for hereditary endocrine tumors. Endocrine (2018) 59:438–48. 10.1007/s12020-017-1322-5 28530019

[5] Goudet P Dalac A Le Bras M Cardot-Bauters C Niccoli P Lévy-Bohbot N. MEN1 disease occurring before 21 years old: a 160-patient cohort study from the Groupe d'étude des Tumeurs Endocrines. J Clin Endocrinol Metab. (2015) 100:1568–77. 10.1210/jc.2014-3659 25594862

[6] Goswami S Peipert BJ Helenowski I Yount SE Sturgeon C. Disease and treatment factors associated with lower quality of life scores in adults with multiple endocrine neoplasia type I. Surgery (2017) 162:1270–7. 10.1016/j.surg.2017.07.023 28919050

[7] Wang W Kong J Nie M Jiang Y Li M Xia W. Primary hyperparathyroidism in Chinese children and adolescents: a single-centre experience at Peking Union Medical College Hospital. Clin Endocrinol. (2017) 87:865–73. 10.1111/cen.13453 28833384

[8] Nilubol N Weinstein LS Simonds WF Jensen RT Marx SJ Kebebew E. Limited parathyroidectomy in multiple endocrine neoplasia type 1-associated primary hyperparathyroidism: a setup for failure. Ann Surg Oncol. (2016) 23:416–23. 10.1245/s10434-015-4865-9 26542588

[9] Keutgen XM Nilubol N Agarwal S Welch J Cochran C Marx SJ. Reoperative surgery in patients with multiple endocrine neoplasia type 1 associated primary hyperparathyroidism. Ann. Surg. Oncol. (2016) 23 (Suppl. 5):701–7. 10.1245/s10434-016-5467-x 27464610 PMC6415766

[10] Fyrsten E Norlén O Hessman O Stålberg P Hellman P Long-term surveillance of treated hyperparathyroidism for multiple endocrine neoplasia type 1: recurrence or hypoparathyroidism? World J Surg. (2016) 40:615–21. 10.1007/s00268-015-3297-9 26541865

[11] Vignali E Viccica G Diacinti D Cetani F Cianferotti L Ambrogini E. Morphometric vertebral fractures in postmenopausal women with primary hyperparathyroidism. J Clin Endocrinol Metab. (2009) 94:2306–12. 10.1210/jc.2008-2006 19401378 PMC3214277

[12] Burgess JR David R Greenaway TM Parameswaran V Shepherd JJ. Osteoporosis in multiple endocrine neoplasia type 1: severity, clinical significance, relationship to primary hyperparathyroidism, and response to parathyroidectomy. Arch Surg. (1999) 134:1119–23. 10.1001/archsurg.134.10.1119 10522858

[13] Lourenço DM Jr Coutinho FL Toledo RA Montenegro FL Correia-Deur JE Toledo SP. Early-onset, progressive, frequent, extensive, and severe bone mineral and renal complications in multiple endocrine neoplasia type 1-associated primary hyperparathyroidism. J Bone Miner Res. (2010) 25:2382–91. 10.1002/jbmr.125 20499354

[14] Coutinho FL Lourenço DM Jr Toledo RA Montenegro FL Correia-Deur JE Toledo SP. Bone mineral density analysis in patients with primary hyperparathyroidism associated with multiple endocrine neoplasia type 1 after total parathyroidectomy. Clin Endocrinol. (2010) 72:462–8. 10.1111/j.1365-2265.2009.03672.x 19650788

[15] Gibril F Schumann M Pace A Jensen RT. Multiple endocrine neoplasia type 1 and Zollinger-Ellison syndrome: a prospective study of 107 cases and comparison with 1009 cases from the literature. Medicine (2004) 83:43–83. 10.1097/01.md.0000112297.72510.32 14747767

[16] Salmeron MD Gonzalez JM Sancho Insenser J Goday A Perez NM Zambudio AR. Causes and treatment of recurrent hyperparathyroidism after subtotal parathyroidectomy in the presence of multiple endocrine neoplasia 1. World J Surg. (2010) 34:1325–31. 10.1007/s00268-010-0605-2 20431882

[17] Tonelli F Giudici F Cavalli T Brandi ML. Surgical approach in patients with hyperparathyroidism in multiple endocrine neoplasia type 1: total versus partial parathyroidectomy. Clinics (2012) 67 (Suppl. 1):155–60. 10.6061/clinics/2012(sup01)26 22584722 PMC3328832

